# Ethics in Transplantation

**Published:** 1992-03

**Authors:** R. M. R. Taylor

**Affiliations:** Consultant Surgeon and Head of Transplantation Royal Victoria Infirmary, Newcastle-upon-Tyne


### Ethics in Transplantation

### Based on a Lecture to the Bristol Division of the B.M.A. 11th December 1991

**R. M. R. Taylor, FRCS,**
Consultant Surgeon and Head of Transplantation
Royal Victoria Infirmary, Newcastle-upon-Tyne

The basic reason why ethical problems have arisen in transplantation surgery is because a series of treatments have been established which are at best lifesaving and at the very worst, totally transform the lives of those who receive a graft. The kidney was the first vascularised organ to be successfully transplanted in the 1950's and the evolution of renal transplantation since then has been partly due to the efficacy of first haemodialysis and then continuous amulatory peritoneal dialysis which allowed patients in renal failure to be kept alive until such time as a suitable graft became available. In the last ten years similarly successful programmes of liver, heart, and heart/lung transplantation have become established.

### ORGAN PRESERVATION

It is now possible, by simple cold storage, to keep a kidney viable for 48 hours, a liver for around 20 hours, a heart for six hours, and a lung for four hours. This preservation time allows an organ to be retrieved from a donor dying at some distance from the centre in which it will be transplanted and, in the case of kidneys in particular, for the donor kidneys to be transported to the best recipient within the United Kingdom. Evolving from this ability to preserve organs is an extensive network of sharing of transplantable organs between Transplant Centres within the United Kingdom. This sharing is administered by the United Kingdom Transplant Sharing and Support Service based in Bristol.

### THE RESULTS OF TRANSPLANTATION

Good kidneys transplanted into good recipients will produce graft survival rates in excess of 80% at one year. The same survival can be expected for heart recipients and liver recipients and perhaps a little less for heart/lung recipients. Successful transplantation in good recipients has led to attempts to transplant patients who, a few years ago, would not have been considered. In renal transplantation, children and infants are regularly transplanted and people in their 70's are not disbarred from receiving a graft if otherwise reasonably fit. The upper age limit for heart and liver recipients keeps increasing. This relaxation of the criteria for accepting a potential recpient has increased the recipient pool and widened the gap between recipient need and donor supply. Recipient demand can sometimes lead to use of donors who are not ideal.

### IMMUNE SUPPRESSIVE AGENTS

Modern immune suppression using cyclosporin, azathioprine, steroids, antithymocyte globulin, and monoclonal antibodies in varying combinations and sequences has produced a series of powerful weapons against the immune response so that a recipient's immune system can usually be suppressed sufficiently to allow retention of the graft and, at the same time, maintain sufficient responsiveness to cope with every day infections. However, rejection remains the major cause of graft loss in the first three months and viral infections can sometimes be fatal.

### THE BASIS OF THE ETHICAL PROBLEM

Table 1 shows the number of renal transplants carried out annually in the United Kingdom alongside the waiting list for this treatment. There are at least the same number of patients again on dialysis in this country who could benefit from a renal transplant if sufficient organs were available but who never get on the waiting list because of the unlikelihood of ever receiving a transplant. The waiting lists for intra-thoracic organs and livers are much shorter because patients accepted onto these lists will usually die within months if no donor organ is found. Out of the huge discrepancy between demand and supply arise all the ethical problems.

| | Kidney Transplants | Waiting Lists |
|---|---|---|
| 1983 | 1182 | 2693 |
| 1984 | 1552 | 2780 |
| 1985 | 1428 | 3443 |
| 1986 | 1586 | 3468 |
| 1987 | 1558 | 3564 |
| 1988 | 1612 | 3684 |
| 1989 | 1837 | 3705 |
| 1990 | 1870 | 3854 |

### LIVER DONOR TRANSPLANTATION

Attitudes to live donor transplantation in this country vary. Some Centres will never do a live donor transplant and in others they comprise 25% of all kidney grafts carried out.[1] If one accepts the premise that it is ever justifiable to injure one person for the benefit of another, then guidelines are required as to when it is permissible. Many would argue that it is the right of an individual if he so wishes to donate one of his kidneys to a loved one provided he is fully informed of the risks and alternatives. It is never ethical to use a minor as a donor. Many would regard it as acceptable to use close relatives as donors such as parents or grown-up siblings. Some of us have grave concerns about organ donation from an adult to a parent on the grounds that the potential life-span of the transplant may be regulated by the life-span of the recipient and those who do regard this as an ethical form of transplant create for themselves a very serious problem in deciding what the upper age limit of the potential recipient should be.

The use of second degree relatives such as uncles, aunts and cousins is acceptable under very closely defined circumstances in that the motivation for the donor needs to be scrutinised very carefully alongside the usual careful health checks.

Unrelated donors have caused the greatest ethical difficulties. many would accept the principle of spouse to spouse transplantation. Some would accept emotionally related donors, but these are the most difficult category of all to assess and it is through the loophole of emotionally related donors that many suspect practices have occured. I would contend that it is impossible to assess an emotionally related donor unless by close personal contact with donor and recipient over many years. Therefore, emotionally related donors from overseas cannot be accurately assessed in this country and I believe that for every genuine pair there are many who are not genuine. For this reason, I do not think emotionally related donors should normally be accepted otherwise the gate opens to the unethical practices of paid and coerced donors; practices which have been condemned by the International Transplant Society and the British Transplant Society.[2,3]

In Chicago and Japan there are programmes of liver transplantation using live related donors and there have been instances of live related donation of lobes of lung. To the best of my knowledge, these practices do not exist in the United Kingdom and I firmly hope that they never will.

### CADAVERIC DONATION

It seems to be a common misconception that brainstem death constitutes an ethical problem which is in some way related to organ transplantation. This is untrue on two counts. Firstly, brainstem death is not a difficult diagnosis to make by those trained in critical care and, secondly, the diagnosis of brainstem death has got nothing to do with transplantation since the diagnosis must be made from time to time to allow ventilation of a cadaver to be discontinued and to prevent fruitless waste of precious intensive care resources.

### RIGHTS OF ORGAN DONOR

**The question of who gets the kidney**

It seems he has no rights — but surely he should have. The carrying of a donor card, the writing of a Will, the telling of relatives of a wish to donate, can all become meaningless at the time of death since none of these things has substance in UK law with regard to the human body. The next of kin occasionally deny the professed wish of the prospective donor — a situation which is surely completely unethical and indeed ought to be made illegal.

### THE RIGHTS OF THE POTENTIAL RECIPIENT

In some ways these are clear. For example, he should expect to receive an organ which has got a good chance of being of great benefit to him. But many ethical questions do exist, how much should the recipient be allowed to know about the donor, how much information is he entitled to ask about the quality of the organ, the grade of the match, the capabilities of the team carrying out the retrieval, and indeed the capability of his own medical attendants? I suggest that some of these rights must necessarily be restricted. I do not believe it would be advantageous to provide a whole profile of donor information lest we face the situation where every potential recipient wants a 20 year old donor with a perfect tissue match and is zero rated for all the risk factors in organ donation. Such a situation would result in a massive waste of organs while potential recipients do their window shopping and decide which they might agree to have. Like so many other situations in clinical medicine, I believe the potential recipient must trust the transplant team.

One interesting dilemma in recipient rights relates to who should decide which potential recipient recieves which donor organ. Traditionally in the UK this has been the responsibility of the transplant surgeon, aided by the advice of the physician managing the recipient. Some hold views, however, that this responsibility is too great for one person — particularly a surgeon! and some attempts have been made at decision by Committeee. These Committees do not work and have been abandoned. In the United States of America, the United Network for Organ Sharing (UNOS) has instituted a system whereby all recipients in the nationwide organisation are allocated points obtained from factors such as the closeness of match between donor organ and recipient tissue type, age, time on dialysis, physical fitness, usefulness to society. Organs are allocated on a mathematical scale. With all its defects, I think the British system has much to commend it.

### OTHER ASPECTS OF ORGAN SHARING

There are many ethical dilemmas under this heading. How should organ sharing be organised between centres? Should it be simply based on securing the best match possible for a donor organ and, if so, how does one avoid huge imbalances through the sharing system whereby one centre makes great efforts to identify donors, retrieve organs and yet sees many of its own patients suffering because too many organs are sent to other centres. Linked with these problems is the argument that exported organs are not usually as good as "home grown" ones because if one of a pair of kidneys is to be sent away, it it never going to be the better of the two. Furthermore, it is known that extended storage times are likely to have a deleterious affect on graft outcome and will increase the cost of looking after the recipient because of delayed function of the graft and longer hospitalisation.

Some thought needs to be given to our very nationalistic attitude to donor organs in that no organ retrieved within the UK may be transplanted into a patient who is not eligible for N.H.S. treatment unless there is no such eligible person for whom it could be used. My own view is that this is a correct position to adopt but some would say that it is very ungenerous.

### FOETAL TISSUE

Is it right to use foetal tissue in transplantation work as, for example, foetal islet cells? I can see no ethical dilemmas here with the following provisos:

(i) the parents informed consent must always be obtained and,

(ii) the foetus must never be conceived or aborted solely for the purposes of tissue donation.

This last comment may appear astonishing but it is already known that in the United States a child was conceived for the sole purpose of becoming a marrow donor for its sibling. While sympathising with the viewpoint of the desperate parents of a dying child, I cannot believe that this is ethical.

### THE GREATEST ETHICAL ISSUE OF THEM ALL

When one sees the benefits that can be obtained from organ transplantation and witnesses the desperate shortage of donor organs, it is unethical for any practising clinician to have under his care a potential donor and not to do everything in his power to ensure that, if the organs can be retrieved, that they are retrieved. It is certainly unethical to disconnect a ventilator without ascertaining the family wishes regarding organ donation. Arguments of causing added stress to relatives are quite invalid when it is known of the great comfort that organ donation can give to relatives both at the time of bereavement and later. The ethical dilemmas, however, are not confined to medical practitioners. The general public also has to resolve its own dilemmas. Each and every one of us must decide with our own conscience how it helps us to have our priceless organs buried or cremated instead of transforming the lives, not only of six different recipients, but of six different families. The gift of a life saving organ is the greatest material gift which one human being can give to another — and it costs the donor absolutely nothing.

### REFERENCES

1. Donnelly P.K., Clayton D.G., Simpson A.R. Transplants from living donors in UK and Ireland — a centre survey. *Br. Med. J.* 1989: **298:** 490.
2. The Council of the Transplantation Society. Commercialisation in transplantation: The problems and some guidelines for practice. *The Lancet.* 1985: **28:** 715.
3. British Transplantation Society. Recommendations on the use of living kidney donors in the United Kingdom. *Br. Med. J.* 1986: **293:** 257.

